# Anatomical relationship between the anterior arm of the semimembranosus tendon and the medial collateral ligament: A cadaveric study

**DOI:** 10.1002/jeo2.70530

**Published:** 2025-11-05

**Authors:** Keishiro Kikuchi, Kosuke Tabuchi, Yoko Tabira, Koichi Watanabe, Georgi P. Georgiev, Joe Iwanaga, R. Shane Tubbs

**Affiliations:** ^1^ Department of Neurosurgery, Clinical Neuroscience Research Center Tulane University School of Medicine New Orleans LA USA; ^2^ Department of Orthopaedic Surgery Kurume University Kurume Fukuoka Japan; ^3^ Department of Anatomy Kurume University School of Medicine Kurume Fukuoka Japan; ^4^ Department of Orthopedics and Traumatology University Hospital Queen Giovanna‐ISUL, Medical University of Sofia Sofia Bulgaria; ^5^ Department of Neurology, Clinical Neuroscience Research Center Tulane University School of Medicine New Orleans LA USA; ^6^ Department of Structural & Cellular Biology Tulane University School of Medicine New Orleans LA USA; ^7^ Department of Neurosurgery and Ochsner Neuroscience Institute Ochsner Health System New Orleans LA USA; ^8^ Department of Surgery Tulane University School of Medicine New Orleans LA USA; ^9^ Department of Anatomical Sciences St. George′s University St. George′s Grenada; ^10^ University of Queensland Brisbane Australia

**Keywords:** anatomy, cadaver, hamstring, surgery, tibial collateral ligament

## Abstract

**Purpose:**

Despite its close anatomical relationship with the superficial medial collateral ligament (sMCL) and deep medial collateral ligament (dMCL), the primary stabilisers of the medial knee, the anterior arm of the semimembranosus (SM) tendon has been scarcely studied anatomically. In addition, there is concern about potential damage to the anterior arm during the tibial cut in total knee arthroplasty (TKA). This study aimed to quantitatively evaluate the anatomical location of the SM's anterior arm and clarify the anatomical relationship with the MCL using gross and histological evaluation.

**Methods:**

Twenty‐two formalin‐fixed cadaveric knees were used. The distance from the joint line to the anterior arm, the anterior arm′s length, and the entry ratio of the anterior arm underneath the sMCL were measured. Four equal‐thickness sections were harvested from the medial tibial condyle, including the anterior arm and MCL, and stained with Masson's trichrome, hematoxylin, and eosin. The anatomical relationship between the anterior arm, sMCL, and dMCL was evaluated.

**Result:**

The vertical distances from the joint line to the proximal and distal margin of the anterior arm are 8.9 ± 1.5 mm and 16.8 ± 1.5 mm, respectively. The entry ratio of the anterior arm underneath the sMCL was 0.53 ± 0.21. The anterior arm fibres intersected with distally extending dMCL fibres. The loose connective tissue was located at the junction of the sMCL, anterior arm, and dMCL.

**Conclusion:**

Anterior arm of the SM tendon shares a common attachment area with the dMCL tibial attachment and is connected to the sMCL via loose connective tissue. The measurements obtained in the present study may serve as useful indicators for avoiding damage to the anterior arm during tibial cut in TKA.

**Level of Evidence:**

Level Ⅳ.

**Abbreviations**
AA: anterior arm
DA: direct arm
dMCL: deep medial collateral ligament
MCL: medial collateral ligament
MM: medial meniscus
POL: posterior oblique ligament
SM: semimembranosus
sMCL: superficial medial collateral ligament
sMCL‐dTA: superficial medial collateral ligament distal tibial attachment
sMCL‐pTA: superficial medial collateral ligament proximal tibial attachment
SMT: semimembranosus tendon
TKA: total knee arthroplasty

## INTRODUCTION

The main structures of the posteromedial knee are the superficial medial collateral ligament (sMCL), the deep medial collateral ligament (dMCL), the posterior oblique ligament (POL) and the semimembranosus (SM) tendon [21, 30, 33]. Among them, the SM tendon is a dynamic stabiliser related to external tibial rotation and anteromedial rotation [2, 18].

The SM arises from a superolateral on the ischial tuberosity and a sacrotuberous ligament. Its primary insertion is the tubercle on the posterior aspect of the medial tibial condyle. The SM distal tendon then distally bifurcates into different areas [35]. LaPrade et al. described eight arms of SM tibial attachments, i.e., (1) direct arm, (2) anterior arm, (3) attachment to the coronary ligament of medial meniscus, (4) lateral tendinous expansion of the main common tendon that contributes to oblique popliteal ligament, (5) proximal posterior capsular arm, (6) distal tibial expansion, (7) oblique popliteal ligament, and (8) components of the POL [20]. Although, there are discrepancies in the reported number and locations of the arms, and the matter remains controversial, the existence of the anterior arm of the SM tendon is in agreement with other previous studies [9, 11, 20, 37]. The main common tendon of the SM bifurcates into the direct and anterior arms at the posterior aspect of the medial tibial plateau. The anterior arm is located within the SM bursa and courses anteriorly (parallel to the joint line) deep to the sMCL [9, 20]. Thus, the anterior arm is believed to have an intimate anatomical relationship with the MCL. According to Warren and Marshall, the anterior arm travels slightly distal to the dMCL tibial attachment [37]. LaPrade et al. stressed that the sMCL has two distinct tibial attachments, i.e., the sMCL proximal tibial attachment (sMCL‐pTA) and distal tibial attachment (sMCL‐dTA). In LaPrade et al.'s study, the sMCL‐pTA is considered to mainly attach to the anterior arm of the SM tendon [21]. Despite its close anatomical relationship with the MCL, one of the primary stabilisers of the medial knee, the anterior arm of the SM tendon has been scarcely studied anatomically.

The anterior arm has a vital role in the biomechanical function of the knee. When the knee is in 90° of flexion, the anterior arm is in alignment with the thigh; it can, therefore, by its action on the tibia, act on the rotations [2, 9]. During the tibial cut in total knee arthroplasty (TKA), there is concern regarding potential damage to the attachments of the ligament that stabilise the knee, such as dMCL and PCL tibial attachment [23, 26]. The anterior arm of the SM tendon is also closely located to the tibial joint surface and has a risk of injury during tibial cut. However, due to the lack of detailed descriptions of the anatomical location of the anterior arm, the risk of injury has not been fully assessed. Therefore, a comprehensive anatomical description of the anterior arm is warranted.

This study aimed to quantitatively evaluate the anatomical location of the anterior arm of the SM tendon and to clarify its anatomical relationship between the anterior arm and the MCL using gloss and histological evaluation. It was hypothesised that the anterior arm of the SM tendon is located slightly distal to the joint line and has a distinct anatomical relationship with the medial collateral ligament complex, which is clinically significant for the tibial cut in TKA.

## MATERIALS AND METHODS

### Preparation and dissection

This is a descriptive anatomical study using formalin‐fixed cadavers. Twenty‐two (eight unpaired and seven paired) Caucasian cadaveric knees were used. All cadavers were donated to the Anatomic Laboratory of the Tulane University School of Medicine for education and research. Cadaveric knees without injuries to the SM tendon or MCL were included. Cadaveric knees with severe osteoarthritis, bone abnormalities, or surgical scars in the dissected area were excluded. All knees were cut approximately 100 mm superior and inferior to the joint line, and skin and subcutaneous tissue were removed. The joint line was defined as the medial tibial plateau. The triceps surae and plantaris muscles were removed. The distal SM tendons were severed about 20 mm proximal to the joint line. The articular joint capsule and the mid‐substance of the sMCL were separated about 10 mm proximal to the joint line to avoid damage to the tibial side of the sMCL. The cruciate ligaments of the knee joint and lateral collateral ligament were divided at the mid‐substance of the ligament, and the femur was removed. The sartorius, gracilis, and semitendinosus muscles were removed at the tibial attachment. After resection of the gracilis and semitendinosus muscles, the sMCL was exposed.

### Gross anatomical observation

**Figure 1:**
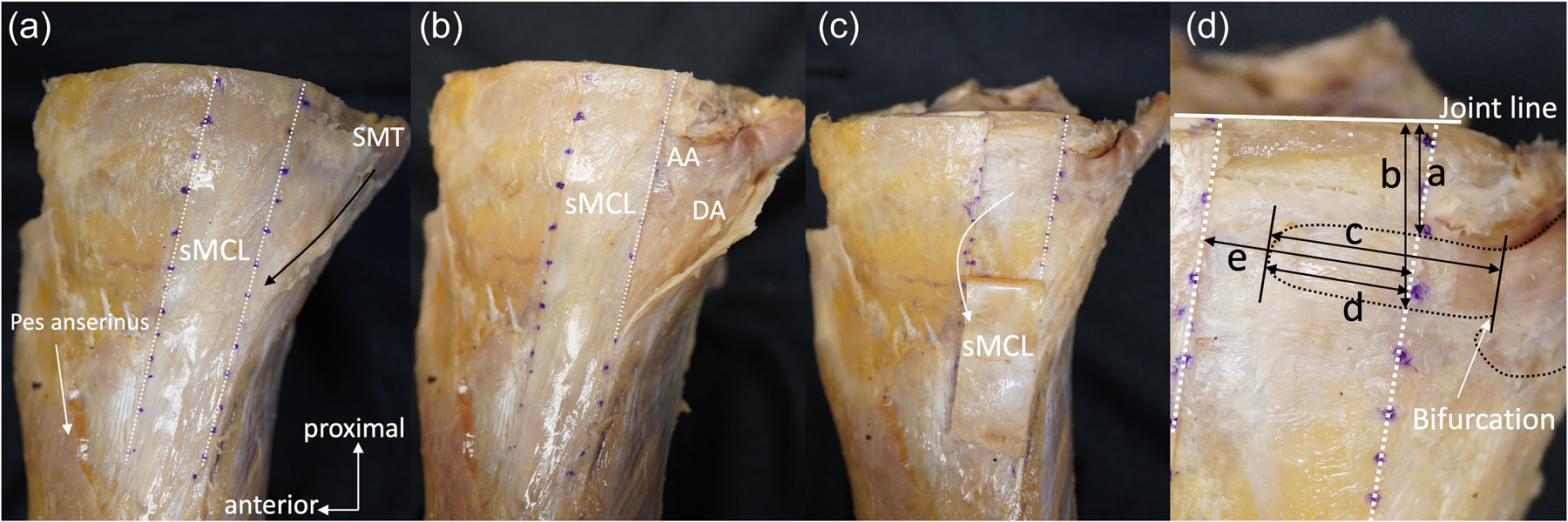
Dissection and gross anatomical measurements of the anterior arm (medial view of the right knee). (a) White dashed lines showing the anterior and posterior margins of the sMCL. The black arrow shows the open cut line of the SM tendon sheath. (b) After the SM tendon sheath resection. The AA and DA are exposed. (c) After the sMCL reflection. Note the AA is fully exposed. (d) The measurements of the AA. a, The vertical distance from the joint line to the proximal margin of the AA at the posterior margin of the sMCL. b, The vertical distance from the joint line to the distal margin of the anterior arm at the posterior margin of the sMCL. c, The distance from the bifurcation of the DA and AA to the anterior margin of the AA. d, The distance between the posterior margin of the AA to the anterior margin of the sMCL. e, The width of sMCL at the level of AA. AA, anterior arm; DA, direct arm; sMCL, superficial medial collateral ligament; SMT, semimembranosus tendon.

Gross anatomical evaluation was performed using fifteen knees (eight from unpaired knees and seven from paired knees; all fifteen knees were harvested from fifteen cadavers). The anterior and posterior margins of the sMCL were identified. The fascial tissue covered the SM tendon, i.e., the SM tendon sheath, was identified posterior to the sMCL. Hughston et al. mentioned that the part of the POL travels just posterior to the sMCL and passes over the SM tendon, which sends fibres to the SM tendon sheath [11]. In this study, it was identified that the fibres travelling obliquely just posterior to the sMCL that appeared to be the POL but could not distinguish these fibres from the SM tendon sheath. The POL and the SM tendon sheath could not be distinguished. The SM tendon sheath was cut and opened obliquely from the proximal side of the SM tendon toward the posterior margin of the sMCL (Figure 1a). The SM tendon sheath was resected, taking care not to damage the SM tendon and posterior margin of the sMCL. The bifurcation of the SM tendon's direct arm and anterior arm was then identified (Figure 1b). The direct arm was attached to the proximal aspect of the posteromedial part of the tibia, and the anterior arm passed anteriorly under the sMCL. The anterior arm was defined as an anterior extension of the SM tendon fibres parallel to the joint line. The sMCL was reflected inferiorly from the joint line to the sMCL‐dTA (Figure 1c). When the sMCL was reflected, the structure between the sMCL and the anterior arm was confirmed. Subsequently, the anterior arm′s proximal, distal, and anterior margins were identified after sMCL was reflected. Then, the meniscotibial ligament was resected, and the medial meniscus was removed to expose the joint line. Finally, the multiple parameters (distance) shown in Figure 1d were measured. The entry ratio of the anterior arm underneath the sMCL, i.e., the ratio of (distance from anterior margin of the anterior arm to posterior margin of the sMCL)/(sMCL width), was also measured. The observer (KK) performed the observations and the measurements.

### Histological observation

**Figure 2:**
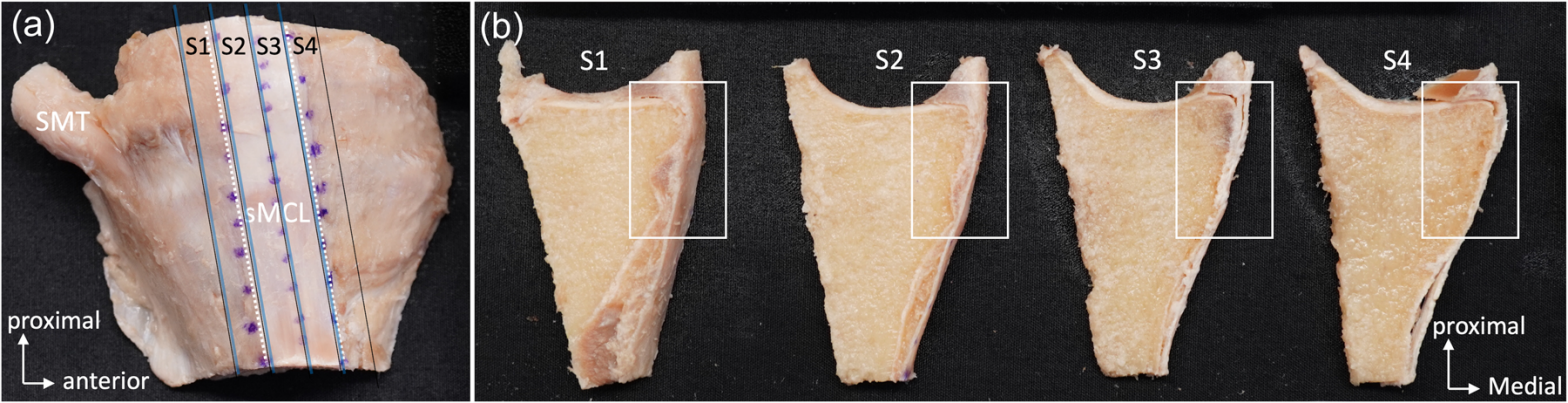
Dissection and histological examination of the anterior arm. (a) Medial view of the left knee. Posterior planes of four equal‐thickness sections (S1–4), shown by blue lines, were used for histological evaluation. Each section has an equal thickness to half of the sMCL width. White dashed lines show the anterior and posterior margins of the sMCL. (b) Posterior view of each section of S1–4. The white rectangular area of each section was used for histological evaluation. sMCL, superficial medial collateral ligament; SMT, semimembranosus tendon.

Histological evaluation was performed using the contralateral side of the seven paired knees used in the gross anatomical evaluation. Four equal‐thickness sections (S1–4) were harvested from the medial tibial condyle, including the anterior arm and MCL with the split line as posterior to the sMCL, posterior margin, midline and anterior margin of the sMCL and anterior to the sMCL (Figure 2). The midline of the sMCL was defined as the line that connects two points: the centre of the sMCL at the level of the joint line and the centre of the sMCL‐dTA. The other split lines were parallel to the midline of the sMCL. The posterior plane of each section was used for histological evaluation. All sections were decalcified using Epredia™ Decalcifying Solution (Epredia, Michigan, USA). After dehydration with ethanol, ethanol removal with the xylene, and paraffin embedding, the sections were cut at 5μm and stained with Masson′s trichrome, hematoxylin, and eosin. The anatomical location of the anterior arm in each section was qualitatively evaluated. The anatomical relationship between the anterior arm, sMCL and dMCL was evaluated. Three observers (KK, JI and RST) performed the observations. Each observer independently evaluated the seven knees histologically, and the three then reviewed their findings together. When discrepancies arose, the observers have discussed them and reached a consensus.

### Statistical analysis

Measurements in this study are presented as means and standard deviations. Although a formal sample size calculation was not performed, all available cadaveric knees were included, and the number of samples in this study exceeded the minimum recommended sample size of 10 for basic cadaveric research [15].

## RESULT

### Gross anatomical observation

**Figure 3:**
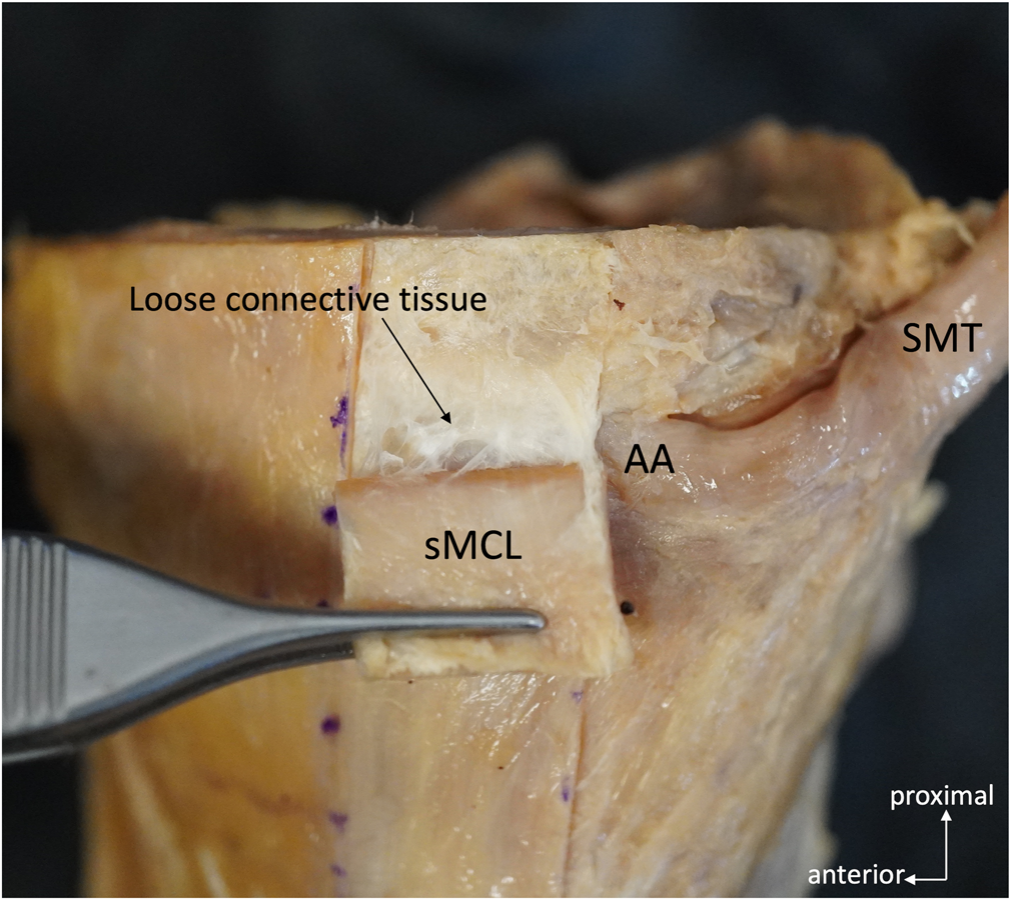
Medial view of the right knee when the sMCL reflected from the joint line to the level of the anterior arm. Loose connective tissue was observed deep in the sMCL. AA, anterior arm; sMCL, superficial medial collateral ligament; SMT, semimembranosus tendon.

Fifteen unpaired knees were used in the gross anatomical observation. The specimens were derived from ten males and five females. The mean age of the donors at the time of death was 82.0 ± 13.0 years (range 58–101 years). When the sMCL was reflected from the joint line, the loose connective tissue was intervened between the sMCL and the anterior arm and tibia. The loose connective tissue was observed slightly distal to the joint line, mainly at the level of the anterior arm, and was present from the anterior to posterior margin of the sMCL (Figure 3). The loose connective tissue was bluntly dissected easily. The anterior arm′s proximal, distal, and anterior margins were exposed after removing the loose connective tissue. The loose connective tissue was observed in all knees. Each measurement from the gross anatomical evaluation is shown in Table 1. The mean distance from the anterior margin of the anterior arm to the posterior margin of the sMCL was 7.2 ± 3.0 mm (3.1–11.6 mm), and the entry ratio of the anterior arm underneath the sMCL was 0.53 ± 0.21 (0.26–0.89).

**Table 1:** Gross anatomical measurements.

Anatomical positional relationship	Mean ± SD (mm)	Range (mm)
Distance from joint line to:		
Proximal margin of the anterior arm (a)	8.9 ± 1.5	6.4–10.8
Distal margin of the anterior arm (b)	16.8 ± 1.5	13.8–18.9
Width of the anterior arm (b‐a)	8.0 ± 1.1	6.1–10.3
Distance from the anterior margin of the anterior arm to:		
Bifurcation of the anterior arm and direct arm (c)	17.3 ± 2.3	13.2–22.3
Posterior margin of the sMCL (d)	7.2 ± 3.0	3.1–11.6
Width of the sMCL (e)	13.7 ± 1.7	10.7–17.3

Abbreviations: SD, standard deviation; sMCL, superficial medial collateral ligament.

### Histological observation

**Figure 4:**
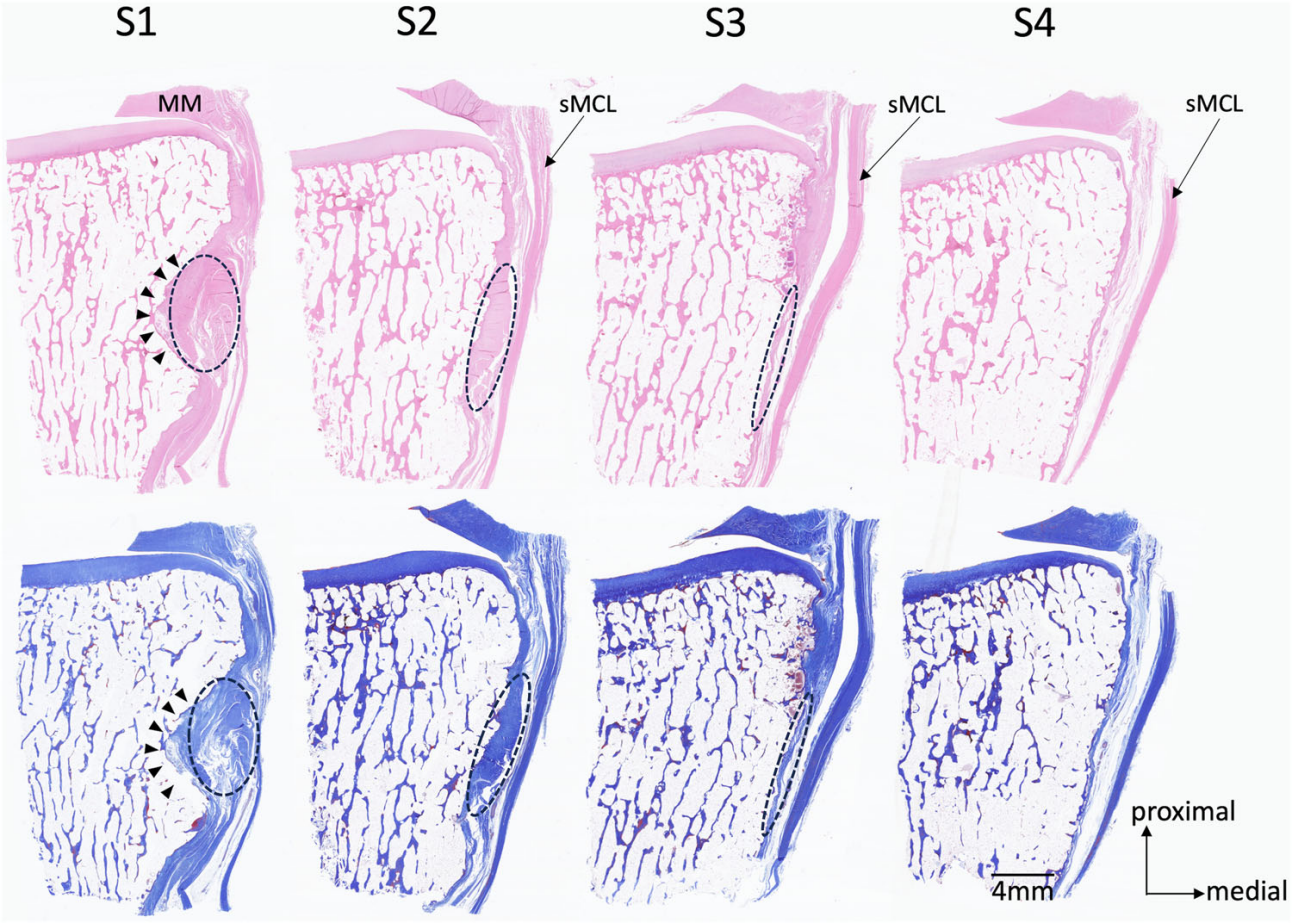
Histological images of the anterior arm. The upper images show Masson's trichrome staining, and the lower ones show hematoxylin and eosin staining. The arrowheads indicate the depression slightly distal to the joint line. A dashed line encloses the area where the anterior arm passes. MM, medial meniscus; sMCL, superficial medial collateral ligament.

**Figure 5:**
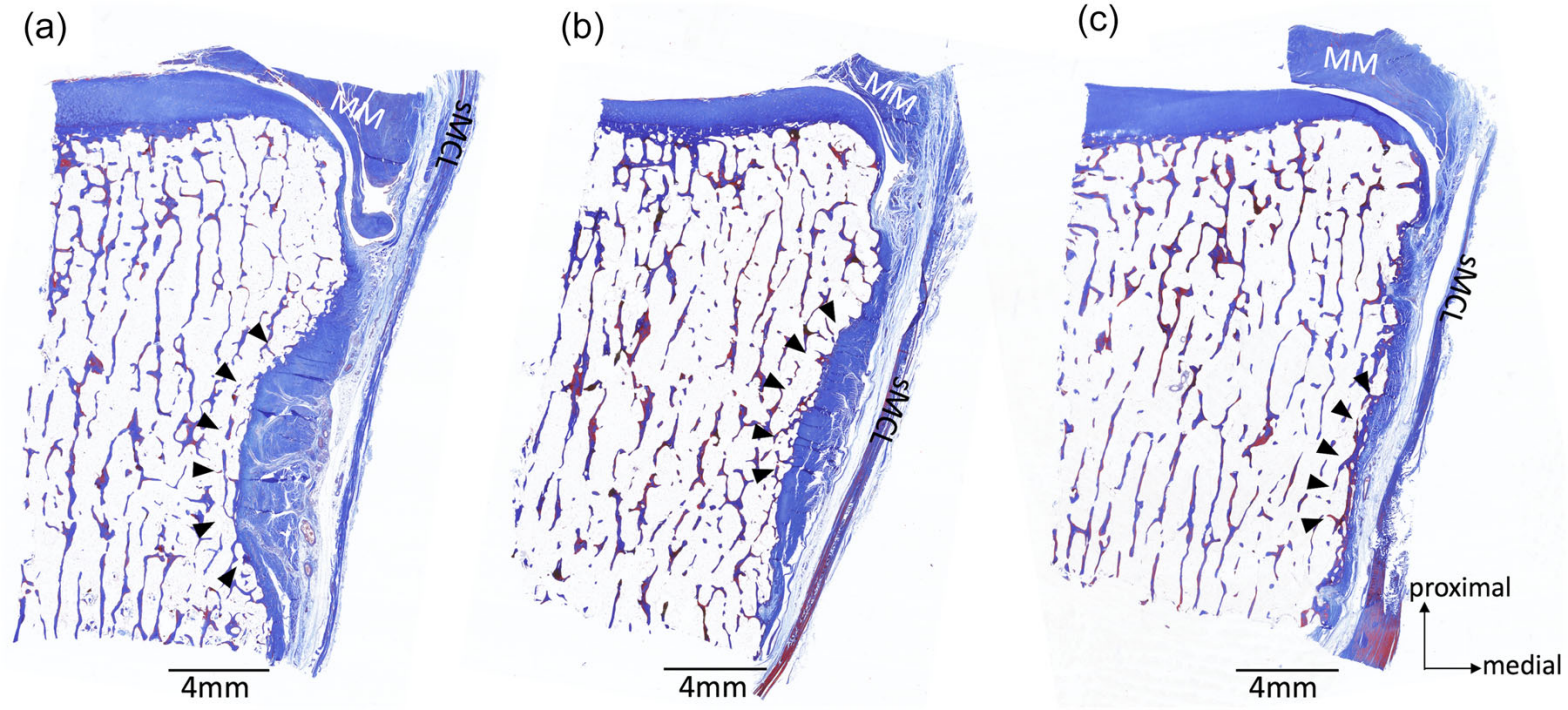
Histological images (a–c) in the S2 section of the three knees. The arrowheads indicate the depression and the anterior arm that passed through it. MM, medial meniscus; sMCL, superficial medial collateral ligament.

**Figure 6:**
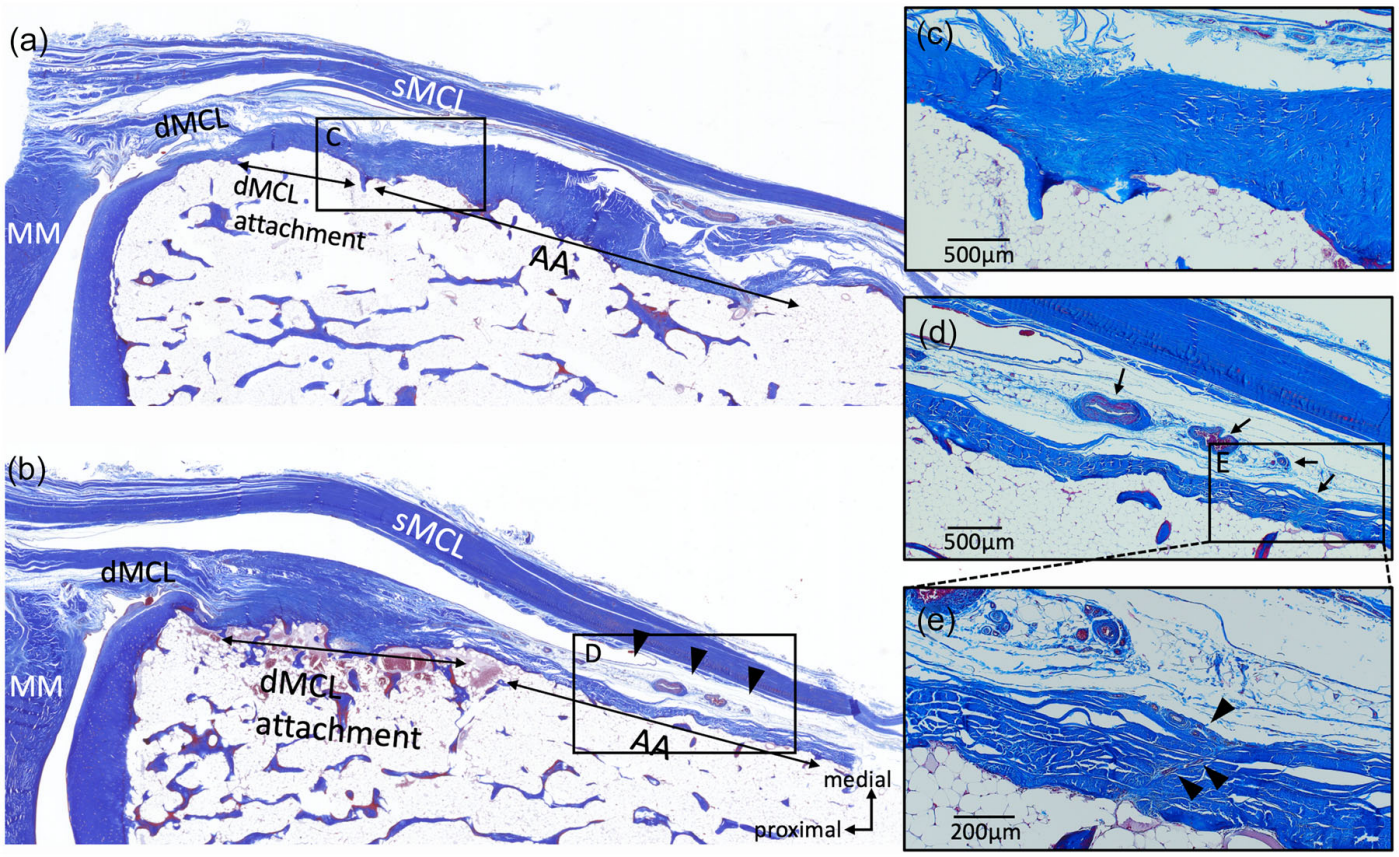
Anatomical relationship of the anterior arm and the MCL. (a) Histological image in the S2 section. The double arrows show the primary attachments of the dMCL and anterior arm. (b) Histological image in the S3 section. The arrowheads indicate the loose connective tissue between the sMCL and the anterior arm. (c) Magnified image of the rectangular area in (a). Some of the dMCL fibres extended and intersected with the anterior arm. (d) Magnified image of rectangular area in (b). The double arrows show the blood vessels in the loose connective tissue. (e) Magnified image of the rectangular area in (d). The arrowheads show the loose connective tissue extending into the anterior arm. AA, anterior arm; dMCL, deep medial collateral ligament; MM, medial meniscuss; MCL, superficial medial collateral ligament.

**Figure 7:**
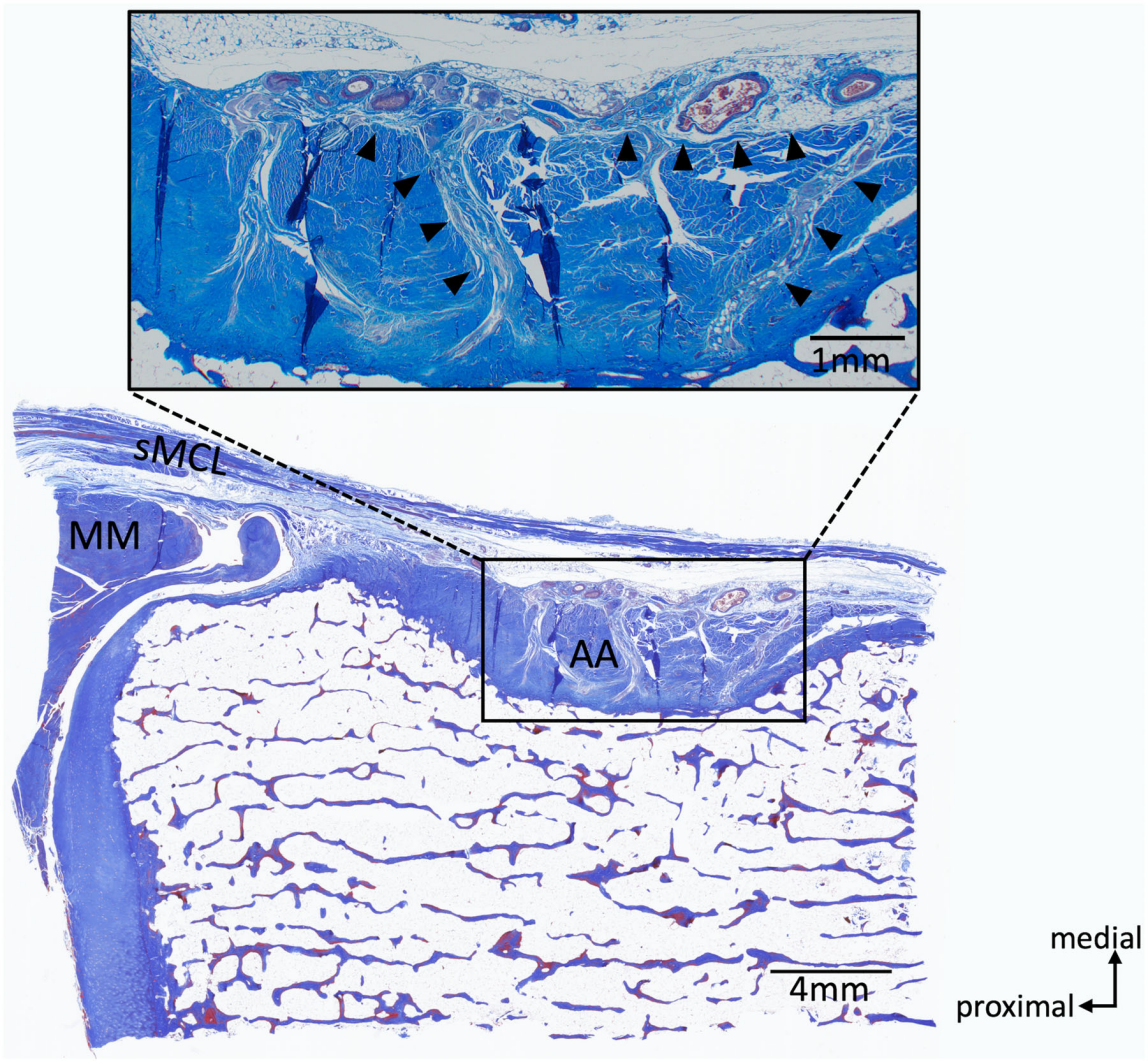
Histological image in the S2 section and magnified image of the anterior arm. The arrowheads show the connective tissue containing blood vessels extending into the anterior arm. AA, anterior arm; MM, medial meniscus; sMCL, superficial medial collateral ligament.

Seven unpaired knees were used in the gross anatomical observation. The specimens were derived from five males and two females. The mean age of the donors at the time of death was 87.3 ± 13.4 years (range 66–101 years). A depression was slightly distal to the joint line, and the anterior arm sat on it. The depression became shallower towards the anterior side of the tibia, and the thickness of the anterior arm became thinner (Figure 4). The anterior arm was observed in all knees of the S1 and S2 sections (100%), in six of seven knees of the S3 section (85.7%), and two of seven knees of the S4 section (28.6%). The depth of the depression and the thickness of the anterior arm at the posterior margin of the sMCL (S2 section) differed largely among the cases (Figure 5). The anterior arm was located slightly distal to the dMCL tibial attachment. The anterior arm fibres intersected with distally extending dMCL fibres (Figure 6). The loose connective tissue was located in the junction of the sMCL, anterior arm and dMCL (Figure 6b,d,e). Blood vessels were constantly observed in the loose connective tissue (Figure 6b,d,e). The loose connective tissue with rich blood vessels was observed on the surface of and within the anterior arm (Figures 6e and 7). These findings were consistent in all knees.

## DISCUSSION

The main finding of the present study was the quantitative clarification of the anatomical location of the anterior arm of the SM tendon. In addition, the anatomical relationships of the anterior arm and sMCL and dMCL were evaluated histologically.

In the present study, the distances from the joint line to the proximal and distal margins of the anterior arm are 8.9 mm and 16.8 mm, respectively. There is always a risk of injury of ligament and tendon attachments around the knee associated with a tibial cut in the TKA for the osteoarthritis of the knee. Maes et al. revealed that the mean distance from the joint line to the dMCL tibial attachment is 4.7 mm and, on average, 54% of the dMCL insertion is sacrificed if a standard tibial cut is selected [23]. The distance from the joint line to the anterior arm is a useful indicator to avoid anterior arm injury in cases that tends to require an increased the medial tibial resection amount, particularly in TKA following high tibial osteotomy. Based on the present study, when the medial tibial cut depth is more than 9 mm, not only the dMCL tibial attachment but also the anterior arm of the SM tendon might be damaged.

In the present study, the anterior arm ran slightly distal to the dMCL tibial attachment, with some dMCL fibres being interdigitated with the anterior arm and extending further distally. The anterior arm and the dMCL distal attachment overlapped in all cases. This study is the first to confirm that the dMCL tibial attachment and anterior arm of the SM tendon are not completely independent separate regions of the tibial attachment, but have a common attachment area. In addition, loose connective tissue intervening between the sMCL, the anterior arm, and the dMCL tibial attachment was identified. Some descriptions of the anatomical relationship between the anterior arm and sMCL exist. LaPrade et al. reported that the sMCL has two distinct tibial attachments, i.e., the sMCL‐pTA and sMCL‐dTA. In the sMCL‐pTA, the sMCL is considered to be mainly attached to the anterior arm [21]. Other anatomical studies supported LaPrade's finding [17, 22, 31]. However, previous anatomical studies were performed macroscopically. The sMCL fibres did not directly attach to the anterior arm in the present study′s histological observation. Further investigation is necessary to determine whether the loose connective tissue can be defined as the attachment site of sMCL. The loose connective tissue had rich blood vessels, with some extending to the anterior arm. Georgiev et al. reported that the thin layer of connective tissue adherent to the sMCL is termed the epiligament, which has rich blood vessels that nourish the ligament [6]. The connective tissue identified in this study was similar to the histological findings of epiligament. This tissue may function not only as a connection between sMCL and the anterior arm, but also as a source of nourishment of the anterior arm. The sMCL and dMCL are important stabilisers of the medial knee [8, 29, 34]. Anatomical knowledge of the relationship between the anterior arm and the MCL will contribute to a better understanding of the complex mechanisms of knee stabilisation.

During the TKA, the importance of achieving proper limb alignment and good soft tissue balancing has been emphasised, and step‐by‐step cutting or release of the medial structures of the knee to gain appropriate soft tissue balancing has been reported by many authors [3, 4, 12, 25, 36, 38, 39]. The SM release is also included in the release of the medial structures of the knee [7, 16, 19]. On the other hand, inappropriate cutting or release of the medial structures of the knee causes instability and abnormal knee kinematics [1, 5, 12]. In this study, it have been clarified the intersection of the anterior arm and dMCL fibres and the loose connective tissue mediated by the sMCL and the anterior arm. Excessive SM release, especially anterior arm release, can affect these structures and cause the native function of the sMCL and dMCL to fail.

Patients with osteoarthritis of the knee or after TKA sometimes experience medial knee pain due to SM tendinopathy or SM tenosynovitis [9, 10]. In these cases, mechanical stressing of the anterior arm from either protruding osteophytes or hardware is thought to cause tendinopathic changes [9, 32]. It has been reported that the surgical treatment of the SM attachment, like drilling of the insertion site and semitendinosus tendon transfer performed for SM tendinopathy, tendinitis, or tenosynovitis that shows resistance to conservative treatment [9, 24, 27, 28].

The anatomical data of the anterior arm presented in this study provide a basis for surgical techniques involving the SM attachment.

The present study has limitations. First, the specimens were predominantly older cadavers due to the nature of anatomical studies. However, this study excluded knees with severe osteoarthritis, bone abnormalities, or surgical scars in the dissected area. Second, due to the limited preservation period of the cadavers, a reliability test was not conducted. Third, no information was available regarding the cadavers' body size (height and body weight). As a result, the correlation between the anatomical finding of the present study and body size could not been verified.

## CONCLUSIONS

Anterior arm of the SM tendon shares a common attachment area with the dMCL tibial attachment and is connected to the sMCL via loose connective tissue. The measurements obtained in the present study may serve as useful indicators for avoiding damage to the anterior arm during tibial cut in TKA.

## AUTHOR CONTRIBUTIONS

**Keishiro Kikuchi**: Research design; data acquisition; analysis and interpretation; writing–original draft; writing–review and editing. **Kosuke Tabuchi**: Research design; data interpretation; writing–review and editing. **Yoko Tabira**: Research design; data interpretation; writing–review and editing. **Koichi Watanabe**: Research design; data interpretation; writing–review and editing. **Georgi P. Georgiev**: Data interpretation; writing–review and editing. **Joe Iwanaga**: Research design; data acquisition; analysis and interpretation; writing–review and editing. **R. Shane Tubbs**: Research design; data acquisition; analysis and interpretation; writing–review and editing. All authors have read and approved the final submitted manuscript.

## CONFLICT OF INTEREST STATEMENT

The authors declare no conflicts of interest.

## ETHICS STATEMENT

This study was conducted in accordance with the requirements of the Declaration of Helsinki (64th WMA General Assembly, Fortaleza, Brazil, October 2013). Our institution does not require the Institutional Review Board (IRB) approval for cadaveric studies as the federal regulations (U.S. Code of Federal Regulation 21 CFR 50.3 (g)). The authors state that every effort was made to follow all international and local ethical laws and guidelines that pertain to the use of samples from human cadaveric donors in anatomical research [14].

## Data Availability

The data that support the findings of this study are available upon appropriate request.
